# Electron relaxation in the CdSe quantum dot - ZnO composite: prospects for photovoltaic applications

**DOI:** 10.1038/srep07244

**Published:** 2014-11-28

**Authors:** Karel Žídek, Mohamed Abdellah, Kaibo Zheng, Tõnu Pullerits

**Affiliations:** 1 Department of Chemical Physics, Lund University, Box 124, 22100, Lund, Sweden; 2 Department of Chemistry, Faculty of Science, South Valley University, Qena 83523, Egypt

## Abstract

Quantum dot (QD)-metal oxide composite forms a “heart” of the QD-sensitized solar cells. It maintains light absorption and electron-hole separation in the system and has been therefore extensively studied. The interest is largely driven by a vision of harvesting the hot carrier energy before it is lost via relaxation. Despite of importance of the process, very little is known about the carrier relaxation in the QD-metal oxide composites. In order to fill this gap of knowledge we carry out a systematic study of initial electron dynamics in different CdSe QD systems. Our data reveal that QD attachment to ZnO induces a speeding-up of transient absorption onset. Detailed analysis of the onset proves that the changes are caused by an additional relaxation channel dependent on the identity of the QD-ZnO linker molecule. The faster relaxation represents an important factor for hot carrier energy harvesting, whose efficiency can be influenced by almost 50%.

Photovoltaics has developed during the last 60 years from “proof-of-concept” experiments to a technology producing about 160 TWh of electrical energy per year (2013). Any further improvement in this field can have an immense effect on costs of energy and the global energy market in general. A great part of research, therefore, nowadays explores various promising concepts of solar cells, among which the quantum dot (QD) based solar cells have a prominent place^1^.

QDs – nanometer-sized semiconductor nanocrystals – can offer a number of merits, such as large absorption cross-section, excellent photostability or tunable absorption spectra^1^,^2^. The tunability enables optimization of the QDs' absorption edge to maximize the solar cell efficiency. The efficiency is, however, even in the ideal case limited to about 30% (single-junction excitonic solar cell) by the so-called Shockley-Queisser limit^3^.

We will describe origin of the limit on the QD-sensitized solar cell, a concept derived from the dye-sensitized solar cells, where QDs serve as an absorber and QD-metal oxide (MO) interface maintains the charge separation by electron injection into the MO.

During photoconversion the absorbed photon excites carriers in QD which consequently relax to the lowest excited state and even further (after charge separation) to MO and electrolyte states. The energy dissipated during the carrier relaxation and extraction is lost for power conversion. Utilizing a part of the energy, which remains unused in conventional methods, would clearly dramatically increase conversion efficiency of any cell.

Several possible concepts for overcoming the losses have been proposed. The most well known is the multiple exciton generation (MEG), where the carriers high above the bandgap use the excess energy to excite the second electron-hole pair. MEG has attracted a huge wave of attention and has been studied for many different QD systems and even solar cells^4^,^5^,^6^,^7^,^8^.

A concept of using a hot electron transfer (HET) is much less studied. The original concept, proposed by Ross and Nozik^9^, consists in extraction of carriers shortly after excitation through a narrow energy band above the lowest energy levels. The excited carriers before complete relaxation can be described by an efficient temperature, which is greater than the lattice temperature of the material. The rapid extraction enables to conserve the excess kinetic energy. A different approach to HET utilization can be found in article of Tvrdy et al.^10^, where authors propose extraction of electrons to different acceptors (different MO materials), depending on their original energy. Nevertheless, both concepts can be realized only for a junction, where a rapid charge separation takes place before the complete carrier relaxation. Parameters affecting the HET efficiency are still unclear and therefore, depending on the particular QD-MO system, published articles report on high efficiency of HET (direct HET measurement of Tisdale et al.)^11^, as well as lack of HET^10^,^12^.

Both HET and MEG (impact ionization) rely on obtaining a slow carrier relaxation in QD compared to the timescale of the corresponding process. Therefore the carrier relaxation is a very important topic for understanding of the hot carrier harvesting in QD-based photovoltaics. Although the carrier relaxation for bare QDs has been subject of many studies^13^,^14^,^15^,^16^, the hot electron dynamics in QD-MO composites has stayed out of focus. Such measurements can be only found as a sideline of some articles without any closer discussion of the observed effects^10^,^11^,^17^.

In this paper we provide a careful study of electron relaxation in three different systems (QD, QDs on SiO_2_ and QDs on ZnO). By comparing the transient absorption (TA) initial kinetics in the three cases, which were analyzed in respect to the onset rate and TA amplitude, we can distinguish changes connected specifically to ZnO attachment. The results enable us to discuss origin of the changes in electron relaxation, especially in respect to the possibility of employing HET and MEG in the QD-MO systems.

## Results

### Steady-state absorption characterization

**Figure 1:**
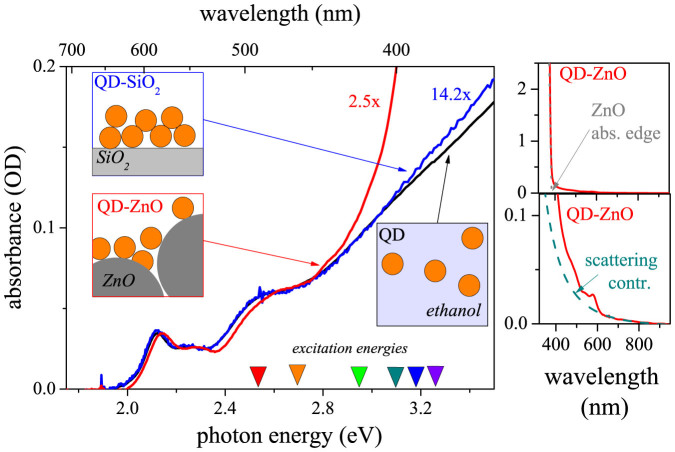
Absorption characterization of the studied samples. Left: Scaled absorption spectra of bare QDs, QD-ZnO (2.5x) and QD-SiO_2 _(14.2x). Spectrum of QD-ZnO was corrected for scattering contribution. Right: Absorption edge of ZnO in the QD-ZnO sample (upper right panel); scattering contribution and the original QD-ZnO spectrum (lower right panel).

The three studied samples are schematically depicted in the insets of Figure 1. QDs dispersed in ethanol (black color) are the simplest system, as they consist of individual non-interacting QDs. Pronounced excitonic features, which can be clearly resolved, imply a narrow distribution of QD sizes in the sample. From position of the lowest excitonic peak 2.12 eV (586 nm) we can estimate the QD size to about 4 nm^18^.

QDs deposited in a thin layer on a flat quartz substrate lead to the same shape of absorption spectra as for the solution sample. The amplitude is, however, significantly weaker due to thickness of the deposited layer. Rough estimate of the thickness can be obtained from absolute OD (0.0029) and the molar extinction coefficient (3.10^5^ cm^−1^M^−1^), both at the lowest excitonic band^18^. This gives us 6.10^13^ QDs/cm^2^ on a flat quartz surface, which corresponds to an ideal coverage of approx. 7 monolayers of QDs. In other words, the quartz sample corresponds to a bulk of aggregated QDs.

Finally, absorption of the QDs-ZnO system consists of the QDs' contribution together with ZnO absorption dominating for wavelengths below 380 nm (see Figure 1, right upper panel). The spectrum is partly distorted by significant Rayleigh scattering (see Figure 1, right lower panel), which can be extracted by fitting λ^−4^ dependence to the spectrum for wavelengths above 670 nm. Here the absorption of QD is negligible (as we know from the solution sample) and the spectrum originates only from scattering.

The absorption spectrum obtained after correction (red color in the main panel) leads to the excitonic features in QD spectra identical to the colloidal QDs. However, all excitonic states are shifted towards blue side. The lowest excitonic state is shifted by 5 nm (18 meV). We point out, that the shift can be observed even without the scattering correction via second derivative analysis. Therefore the correction cannot be the origin of the shift. We will closely discuss origin of the shift later in context of other results.

### Cold electron injection

We used TA to determine charge dynamics in QDs in each of the sample. TA dynamics of CdSe QDs consist of two dominating contributions. The first one is connected to the so-called biexcitonic shift^19^. The excited QD has energy states (and therefore also absorption spectrum) slightly shifted towards red compared to the QD in the ground state. The shift leads to a TA signal similar to the first derivative of the QD absorption spectrum.

**Figure 2:**
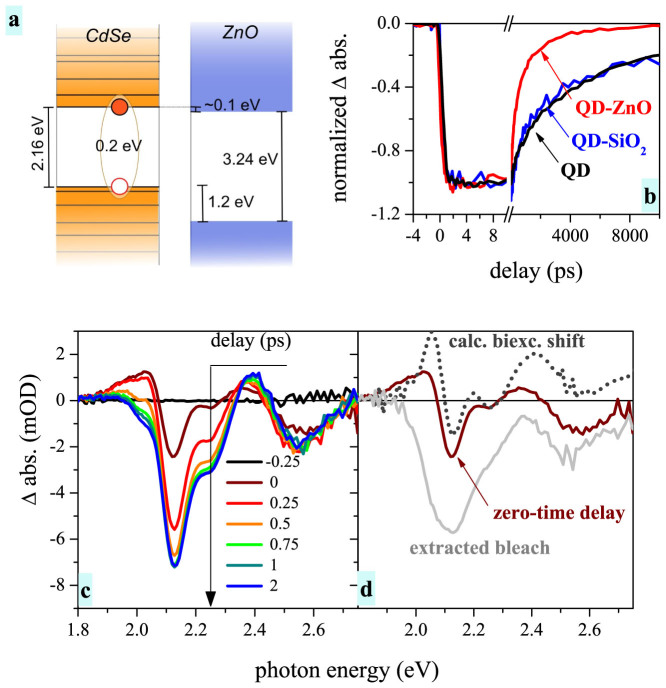
Cold electron injection and its contribution in transient absorption signal. (a) Scheme of energy states alignment and Coulomb electron-hole interaction based on absorption spectra and published results. (b) TA signal decay on the long time scales for all studied samples with a detail of initial dynamics. (c) TA spectra of QDs in solution at various delays (exc. wavelength 390 nm) (d) Zero-time TA spectrum (solid brown line) compared to the spectrum obtained by a 5-meV biexcitonic shift (thin dotted line). Pure TA bleach extracted as a difference between 2-ps and calculated biexcitonic shift (solid grey line).

The shift-related signal appears immediately after excitation, whereas for excitation high above the bandgap the second contribution (bleach) appears only after carrier relaxation. The shift-related signal therefore dominates for the zero-time delay (see Figure 2d). To verify its origin we have shifted the measured steady-state absorption spectrum of QDs by 5 meV towards red^19^. The resulting difference between the shifted and original curve (dotted line) reproduces well the measured zero-time signal even for the amplitude.

The second TA contribution is a bleach of absorption due to state filling of the lowest excited electron state 1S_e_. The state is, according to the effective mass approximation, only doubly-degenerated and its occupation is responsible for the dominating band in the TA spectra at 2.18 eV (corresponds to the lowest absorption band). The bleach can be therefore used to track down electron population in the lowest excited state and provide us with information about electron relaxation.

Before we will investigate the hot electron dynamics it is essential to obtain information about the “cold” electron injection from the 1S_e_ state. Energy band alignment depicted in Figure 2a was obtained from absorption spectra (band gaps of QDs and ZnO) and experimental work of Carlson et al. for the QD-ZnO band alignment^20^. The size of the QDs was chosen so, that the 1S_e_ state is slightly (~0.1 eV) above the conduction band of ZnO. In combination with relatively strong exciton binding energy (0.2 eV for 4-nm-sized QDs)^21^ we have a system, where electron has to overcome a Coulombic barrier before it is injected into ZnO. Therefore the “cold” electron injection is expected to be relatively slow and it should not interfere with the carrier relaxation.

The TA signal in the case of colloidal QDs, as well as QD-SiO_2_, is formed by long-lived bleach decaying due to electron-hole recombination in QDs. The “cold” electron injection should manifest itself as an additional component in the TA decay of QD-ZnO sample. We observe the additional pronounced decay on the nanosecond timescale, which changes the mean lifetime from 6.8 ns for solution to 1.6 ns for the QDs attached to ZnO (see SI for the fit details). This change is in line with measurements of Robel et al. for QD-size-dependent electron injection^22^.

We cannot fully prove that the additional decay in the QD-ZnO sample arises only due to the electron injection, because the decay time is relatively long and other processes can already take place here. Nevertheless, the main point of the long-lived TA decay measurement is to prove absence of any rapid decay during the first 10 ps for any of the measured samples (see Figure 2b). We can therefore study the hot electron dynamics on the time-scale up to 5 ps and safely assume that there is practically no “cold” electron injection taking place in this time range.

### Transient absorption onset dynamics

**Figure 3:**
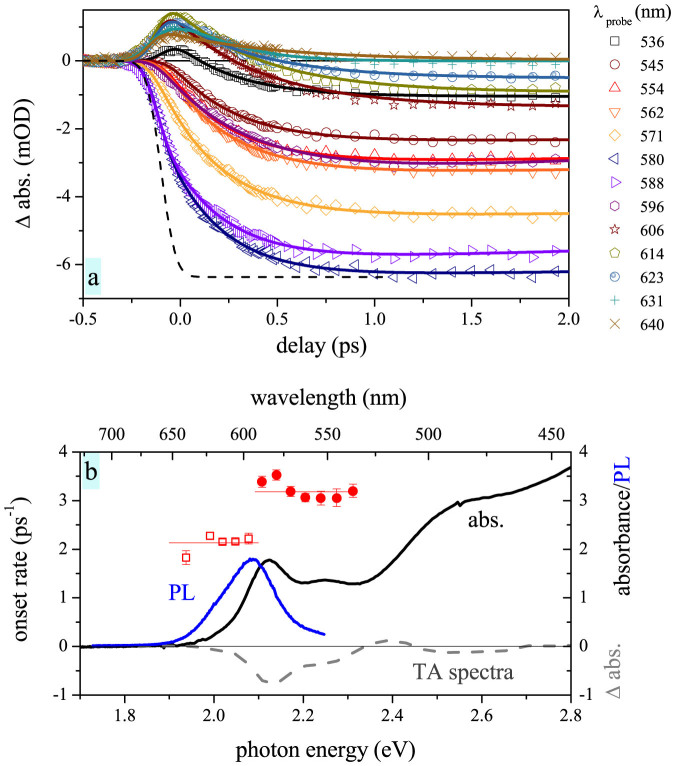
Transient absorption signal fitting showing two distinct spectral regions of onset rates. (a) Dynamics of TA onset for QDs in solution at various probe wavelengths with 3.18 eV (390 nm) excitation. Each kinetics (symbols) is fitted by an exponential rise convoluted with a response function (thick lines) – see text for details. Response-function-limited rise (dashed line) is added for comparison. (b) Onset rates resulting from the fits (symbols), compared to absorption spectrum (solid black line), PL spectrum (solid blue line) and TA spectrum at 2 ps delay (gray dashed line).

We will now focus closely on the initial TA signal. As we discussed before, TA dynamics of CdSe QDs consist of two dominating contributions: bleach of absorption due to state filling and shift of absorption due to the so-called biexciton shift. Whereas the bleach is present only after the electron relaxes into the lowest state, the biexcitonic shift is an immediate response of the QD. To take into account both components each curve was fitted by an exponential rise (rate *k*_R_) combined with a consequent decay (rate *k*_D_), which account for the dominating bleach. The second immediate component is included without any exponential rise: 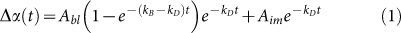
*A*_bl_ and *A*_im_ denote the amplitudes of the dominant bleach and immediate component, respectively. The function in equation (1) was convoluted with Gaussian response function 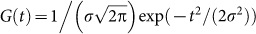, where the response distribution width (FWHM 120–160 fs) was set from measurements of Kerr effect in a thin quartz plate. The fit is an approximation assuming that a single relaxation time can describe the electron relaxation. We demonstrate in SI that this approximation can be used for our system. This approach is also supported by the fact that we obtained a very good agreement between the onset data and the fit (see Figure 3a).

By fitting kinetics at each wavelength we obtain a spectral dependence of the relaxation rates. The rates are depicted on Figure 2b, together with corresponding absorption, PL and TA spectrum. Apparently, two distinct spectral regions of relaxation rates can be observed (red open squares and filled circles in Figure 3b). Those are delimited by the energy of approximately 2.1 eV.

**Figure 4:**
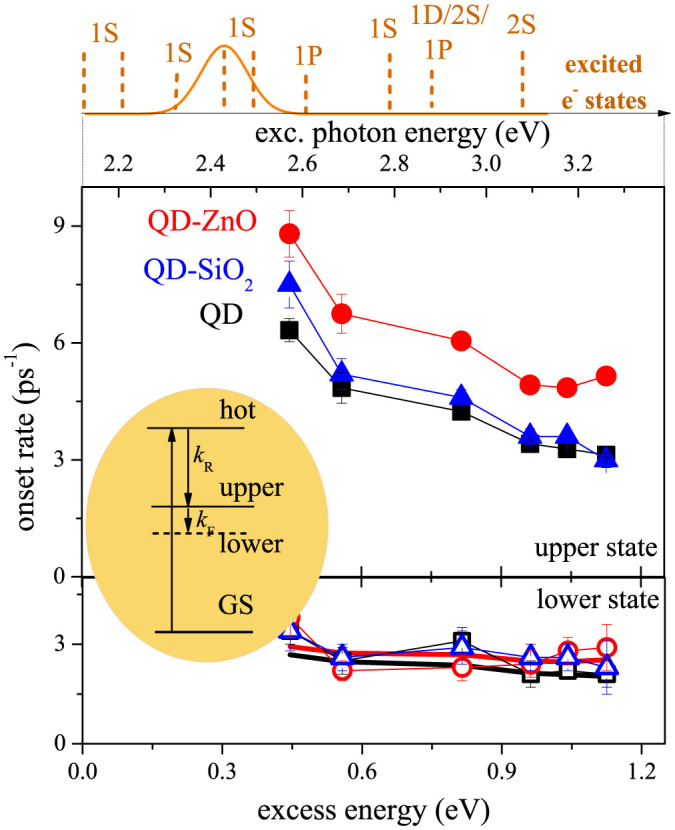
TA onset rates for various excitation photon energies. Upper line: Electron states involved in optical transition studied in our samples – adapted from Ref. 24. Gaussian distribution at transition at 2.45 eV denotes inhomogeneous distribution of energy states. Inset: scheme of excitonic states involved in carrier relaxation (hot – hot excited state, upper/lower – upper/lower excitonic states in fine splitting, GS – ground state). Main panels: Onset rate for the population in 1S_e_ state (upper excitonic levels in upper panel, lower excitonic level in lower panel) under various excitations (exc. photon energies). QDs in solution (black squares), QDs on SiO_2_ (blue triangles), and QDs attached to ZnO (red circles). Solid lines (red for QD-ZnO, black for QD) denote expected lower state onset rate – see text for more details.

It might be surprising that the lowest states, connected to the same 1S_e_ electron state, show two distinct TA onset rates. Effect of the higher electron states (for instance 1P_e_) can be excluded, as they are involved only in transitions above 2.6 eV. This is energetically very far compared to the inhomogeneous broadening in our samples (see Figure 4, upper scheme).

The observed behavior can be explained by using an excitonic picture of the energy states in QDs. So far, we have considered electron states without taking a hole presence into account. However, electron-hole exchange interaction causes splitting of the e-h levels into the so-called exciton fine structure (hexagonal symmetry of the QD lattice plays a role here as well)^19^. Previously it has been shown that simplification of the fine structure into two levels describes well the QD properties^19^. We will denote the states as “upper” and “lower”, according to their energy.

As the upper state features higher dipole moment, it contributes more to absorption spectra. The lower state is, on the other hand, dominating the PL emission^19^,^23^. Indeed, the two spectral regions of the lower (open squares) and higher (solid circles) onset rates correlate with the spectral region of the pronounced PL emission (blue line in Figure 3b) and absorption (black line), respectively.

### Relaxation rates to the lowest excitonic levels

We have systematically carried out analysis depicted in Figure 4 for the three studied samples (QD, QD-SiO_2_, and QD- ZnO) and 6 excitation photon energies. In each case we observed distinction of TA onset rates into two spectral regions, likely corresponding to the upper and lower excitonic levels in fine structure of QD energy states. We have therefore extracted the rate of electron relaxation to both (“upper” and “lower”) excitonic states of the 1S_e_-1S^3/2^_h_ transition.

We can observe quite different behavior for the two states. We will start by discussing the relaxation into the upper state, which clearly differs for the three studied samples. Compared to the reference case of QDs in solution, deposition of QDs on SiO_2_ leads to a very slight increase in the relaxation rate. On the other hand, attachment of QDs to ZnO significantly speeds up the TA onset. This is interesting as the change occurs only for MO with the possibility of charge separation. We will discuss origin of the change in detail in the next sections.

When we turn to the TA onset rate of the lower excitonic state, it does not differ (within our error) from sample to sample and it stays almost constant for all excitation wavelengths. This suggests, that we observe a two-step relaxation (see inset of Figure 4), where the relaxation between the upper and lower states (rate *k*_F_) represents the bottleneck and determines the relaxation time into the lower state. Our results can be reproduced very well by assuming a presence of the second relaxation step with a rate of 3 ps^−1^ (thick solid lines in Figure 4 lower panel, see SI for more details).

The obtained rates for QDs in ethanol agree with values previously measured for CdSe QDs. The 1P_e_-1S_e_ electron relaxation rate has been previously reported as 6.3 ps^−1^ for the 4 nm sized of QDs (compare to 6 ps^−1^ rate at 2.6 eV excitation)^14^. The second step, likely corresponding to relaxation within the fine excitonic structure, has been studied extensively for the pure QDs^25^. The rate for our size of QD was measured to be 2 ps^−1^ (TOPO-capped QDs) – compare to 3 ps^−1^ in our case^25^.

### Carrier relaxation changes vs. hot electron transfer

Important information attained by the rate analysis is the fact that TA signal onset speeds up after the QD is attached to ZnO. As we will show later, this has consequences for hot electron harvesting in the QD-sensitized solar cells. We will now focus on origin of the changes. The changes appear only in the QD-ZnO system. This is the only case from the three studied samples, where charge separation takes place. This suggests that the changes might be connected to charge separation of the hot carriers (i.e. hot electron transfer, HET).

Presence of HET cannot be directly observed as a component in the TA kinetics. We can, however, test the HET presence by analysis of TA onset rate and amplitude, as we will demonstrate hereafter. We will start with a simple electron relaxation in a QD with no electron injection (QD and QD-SiO_2_ systems). Electron relaxation from the hot electron level (population *N_H_*) into the upper excitonic level (population *N_U_*) with a rate *k*_R_ can be described for the pure QD system as: 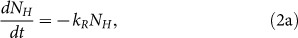

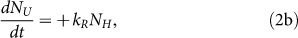


The simple set of equations, which describes the situation when every hot electron relaxes to the upper level has a solution of: 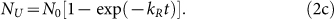
The equations become slightly more complicated in the case of competing electron relaxation and HET (rate *k_I_*). In this case we obtain similar set of equations: 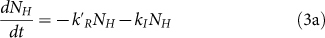

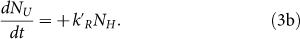


We denote relaxation rate in this case as *k*′*_R_*, because the relaxation rates do not have to be in principle equal in different QD systems. Solution of equations (3a–b) is similar to equation (2c): 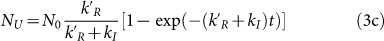
It is, however, worth noting that the apparent relaxation rate and the number of the relaxed electrons (the prefactor of the rising component) have changed.

**Figure 5:**
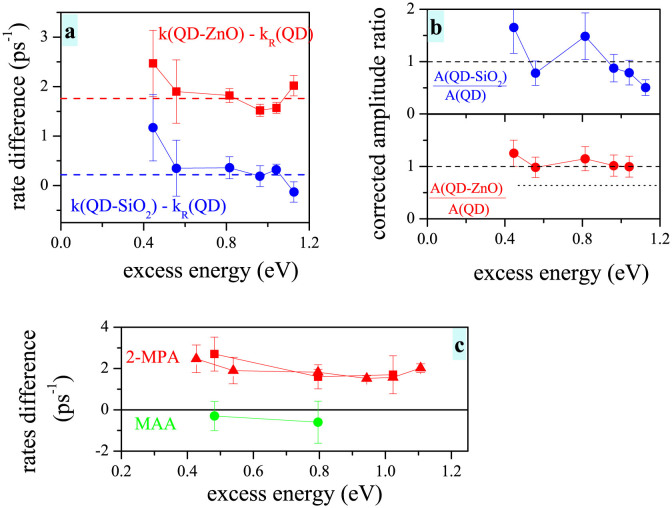
Changes in TA onset rates and amplitudes after deposition of QDs on SiO_2_ and ZnO. (a-b) TA onset rates difference (panel a) and amplitudes ratio (panel b) for QD-SiO_2_ (blue symbols) and QD-ZnO (red symbols) samples. The QDs in solution serve as a reference sample. (c) Comparison between different linker molecules. Different red symbols correspond to sets of measurements on different batches of samples with the same parameters.

We used the isolated QDs in solution as our reference sample and calculated difference in the relaxation rates observed in the other two samples (see Figure 5a). We start our discussion for relatively low excess energies (energy above the QD bandgap) close to 0.4 eV, where we mostly excite 1S_e_ electron state and states energetically close. Here it is expected to observe very little or no HET. We would therefore expect to obtain for all samples a similar TA onset rate. This is, however, not the case, because already here the onset for QD-ZnO is faster by about 2 ps^−1^.

When we turn to the excitations high above the QD bandgap, a presence of HET should intuitively lead to more pronounced differences between the TA onset rates. Instead we observe that the difference of rates in the QD-ZnO system and bare QD stays the same for all excitation energies (within error bars of our experiment).

This behavior, however, does not completely exclude the HET presence. It can be a signature of HET from an intermediate state in the relaxation pathway, which is energetically below 2.6 eV (the lowest studied excitation energy).

The question of HET presence can be decided by employing an analysis of the TA amplitude. The HET should manifest itself in a relative decrease of the TA signal for the QD-ZnO sample. For the measured change in TA onset rate (2 ps^−1^) the TA signal should be reduced by approximately 40% compared to the bare QDs. Instead the amplitude ratios for samples QD-ZnO/QD and QD-SiO_2_/QD (corrected for the sample OD and excitation losses) are within error limits for all excitation energies close to 1, ruling out the HET contribution in our case. Based on the error bars of our TA rate measurements, we can set upper bounds of HET in our system to be approx. 1 ps^−1^ rate, i.e. HET is in our case relatively inefficient, posing upper limitation on HET efficiency to approx. 15%.

### Origin of the relaxation changes

By excluding the HET contribution we can conclude that in the case of the QD-ZnO system we observe changes in electron relaxation induced by QD attachment to ZnO. ZnO surface can act here both indirectly (oxidation of QDs, changes in linker molecules, changes in electron-hole coupling for Auger-type of recombination) and directly (fast hot electron injection into ZnO followed by a rapid back transfer of electron into QD).

The TA amplitude analysis cannot distinguish between different mechanisms of relaxation, or whether the ZnO states are directly involved in the relaxation. To obtain a clearer picture of the processes, we compared measurements for two linker molecules between the QD and ZnO.

All the results presented so far were measured for the QDs connected to ZnO via 2-MPA (2-mercaptopropionic acid) molecule. We have prepared the same system, where we use MAA (mercaptoacetic acid) instead of the 2-MPA. From the “cold” electron injection the MAA linker leads to approximately 3-times faster electron injection^26^. This means that the coupling between the QD and ZnO is in this case significantly stronger. If we assume that ZnO states mediate the faster electron relaxation, we should observe for the MAA linker even more pronounced effect of the QD deposition on the relaxation rate.

Instead, as it is illustrated in Figure 5c, we observe that the relaxation rates in the QD-MAA and QD-MAA-ZnO systems are, within experimental error, the same. In other words the electron relaxation in MAA-capped QDs does not change even after QD attachment to ZnO. We have reproduced the results on different samples using the same linker and QD size (see red squares and triangles in Figure 5c). We have also verified that the TA onset for the 2-MPA linker speed up after attachment to ZnO also for a different QD sizes (1.3 ps^−1^ difference for QDs sized 6.6 nm).

We can therefore exclude the direct involvement of ZnO states in the faster electron relaxation. The striking difference between the relaxation in case of different linker molecules implies that the new relaxation channel is connected to changes on the QD surface induced by attachment to ZnO.

An indication of the changes in QD is also the blue shift in the steady-state absorption spectrum shown in Figure 1. A similar shift is commonly observed phenomenon in various QD-MO composites^27^,^28^,^29^,^30^,^31^. Origin of the shift is still subject of debates.

The partial oxidation of QDs' surface is a straightforward way to explain the blue shift and it is likely one of the factors in our case. The spectral shift of absorption by 5 nm corresponds to decrease in the mean effective QD size by 1.7 Å^18^. Nevertheless, the QD oxidation is typically connected with significant broadening of excitonic features in absorption spectra^31^. We have verified that an analogous blue shift induced by oxidation (photodegradation) of the CdSe QD in the QD-ZnO system leads to apparent broadening of the lowest absorption band (data not shown here), unlike in this case (see Figure 1). This suggests that the QD oxidation is not the only origin of the shift.

We propose a different mechanism which can shift the QD absorption spectrum after attachment to ZnO: alignment of energy of linker HOMO (highest occupied molecular orbital) and LUMO (lowest unoccupied molecular orbital) to QD energy levels. The full calculation using the effective mass theory can be found in Supporting Information; here we will only shortly discuss the results.

The alignment of energy levels between QD and linker can change significantly after linker attachment to MO, because the linker orbitals directly couple to the MO and, at the same time, QD-MO Fermi levels have to be aligned. The linker energy levels form a barrier confining the electron and hole inside the QD. A shift of the linker energy levels should, naturally, lead to a change in carrier confinement in QD, i.e. position of the lowest absorption band.

Our simple calculations show that shift of the linker energy levels by 1 eV towards vacuum level leads to increase in the band gap by 15 meV. The opposite shift, on the other hand, can lead to a red shift. The alignment of the linker level can therefore also play a significant role in the observed changes in the steady-state absorption.

## Discussion

Motivation to study the QD-MO system consist in its application in QD-sensitized solar cells^1^. We will shortly summarize the main implications of the observed relaxation changes.

Processes, such as MEG and HET, aim on energy harvesting before a complete carrier relaxation. Their efficiency is therefore determined to a big extent by the carrier relaxation rate. We will now illustrate the effect of carrier relaxation changes on an example of potential MEG harvesting. By comparing the rate of impact ionization (*k*_MEG_) exciting multiple excitons and relaxation rate (*k*_R_) the efficiency of the hot carrier harvesting can be simply calculated as: 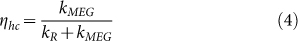
As we have shown in equation (3), the observed TA onset rate represents in fact the sum of rates of both relaxation and hot electron harvesting processes (MEG or HET). The TA measurements allow easily to measure TA onset rate of QD-ZnO system (*k_TA_*) as well as to determine change in relaxation rate induced by attachment to ZnO (Δ*k_TA_*). From the two values it is possible to calculate the relative change in hot carrier harvesting efficiency: 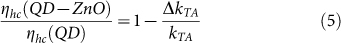
According to our results and equation (5), depending on the used ligand molecule, we can obtain the MEG efficiency unchanged after attachment to ZnO (Δ*k_TA_* = 0 ps^−1^, MAA linker) or decreased by at least 40% (Δ*k_TA_* = 2 ps^−1^, *k_TA_* < 5 ps^−1^ 2-MPA linker). It is worth stressing that such change is present for two linkers, which are otherwise almost identical—the linkers differ only by an additional side –CH_3_ group in 2-MPA.

Our results have two important implications. Firstly, analysis of TA amplitude or TA dynamics separately cannot fully decide between presence of HET or faster relaxation. Simultaneous changes in both have to be present to fully support one or the other scenario.

Secondly, deposition of QDs on metal oxide can significantly speed up carrier relaxation in the QD and this effect is dependent on exact linker molecule. A slight change in linker molecule can lead to a decrease in MEG efficiency by more than 40% after the QD is attached to metal oxide.

This implies that the MEG efficiency might not be conserved in the QD-MO system and a careful selection of QD surface chemistry is needed to achieve an efficient hot carrier harvesting.

## Methods

### Preparation and characterization of samples

Oleic acid-capped CdSe QDs were synthesized using previously reported methods^32^,^33^. Briefly, the trioctylphosphine-Se solution was swiftly injected into the Cd^2+^ precursor solution (mixture of octadecene, oleic acid and CdO), which was heated up to 260°C or 280°C (for QDs with the diameter of 4 nm or 6.6 nm). After 2 min of reaction, the flask was removed from the heater and cooled down to room temperature. As-prepared QDs were purified twice before sensitization.

For the sensitization, the surface capping of the QDs was exchanged from oleic acid to a bifunctional linker molecule (2-mercaptopropionic acid, 2-MPA; mercaptoacetic acid, MAA)^33^. If not stated otherwise, the 2-MPA linker was used in the sample.

ZnO nanowires were synthesized by using a hydrothermal growth on a seeded substrate (fused silica)^34^. The growth was carried out at 92.5°C for 4 h in aqueous solution containing 20 mM hexamethylenetetramine and zinc nitrate. After the growth, the sample was carefully rinsed in deionized water and dried at 100°C.

Finally, the ZnO nanowire films were sensitized by immersing them in the QD solution for 2 h in the dark.

### Transient absorption

Transient absorption signal was recorded using the pump-probe setup described in our previous study^35^. Laser pulses (800 nm, 80 fs pulse length, 1 kHz repetition rate) were generated by a regenerative amplifier (Spitfire XP) seeded by a femtosecond oscillator (Tsunami, both Spectra Physics).

Excitation pulses at the wavelength of 380, 390, 400, 420, 460, and 480 nm were acquired using an optical parametric amplifier (Topas C, Light Conversion). The used excitation photon flux at 480 nm was 1 × 10^14^ photons/cm^2^/pulse corresponds to <*N>* ~ 0.1 (the mean number of excited e-h pairs per QD). The intensity for all excitation wavelengths was adjusted to lead to approximately the same TA signal of the colloidal sample of 5–7.10^−3^ change in OD (measured in 1-mm static cell).

Probe pulses (broad supercontinuum spectrum) were generated from the 800-nm pulses in a sapphire plate and split by a beam splitter into probe pulse and a reference pulse. The probe pulse and the reference pulse were dispersed in a spectrograph and detected by two diode arrays (Pascher Instruments). The analysis of kinetics was done by averaging over 8 nm wide spectral bands of the probe spectra.

Thin film samples (typical OD of 0.02 at first exciton peak) were measured in a nitrogen atmosphere to avoid possible oxidation of QDs^36^.

### Steady-state spectroscopy

Steady-state absorption spectra were measured in the absorption spectrophotometer Agilent 845x. Steady-state photoluminescence of the QD solution was measured using standard spectrometer Spex 1681 with excitation at 470 nm.

## Supplementary Material

Supplementary Information Supporting Information
