# Supplementary material for: Electron relaxation in the CdSe quantum dot - ZnO composite: prospects for photovoltaic applications
Source: Sci Rep. 2014 Nov 28;4:7244. doi: 10.1038/srep07244 (PMC5384232; doi:10.1038/srep07244)
Supplement: Supplementary Information — Supporting Information [file srep07244-s1.pdf]

Supporting information

Electron relaxation in the CdSe quantum dot - ZnO composite: prospects for photovoltaic applications

Karel Žídek,[a] Mohamed Abdellah,[a,b] Kaibo Zheng,[a] Tõnu Pullerits[a]

[a] Department of Chemical Physics, Lund University, Box 124, 22100, Lund, Sweden

[b] Department of Chemistry, Faculty of Science, South Valley University, Qena 83523, Egypt

Table of Contents

1. Losses in QD-based solar cells 1
2. Linker as a finite barrier 2
3. Cold electron injection analysis 4
4. Onset rate analysis 5
5. Effective onset time approximation for two-step relaxation 6
6. Pump-probe amplitude correction for losses 7

1. Losses in QD-based solar cells

Quantum dots (QDs) in QD-sensitized solar cells work as an absorbing material. This suggests that the QDs should absorb as broad range of spectra as possible, i.e. QDs' bandgap should be shifted far into the IR region. On the other hand the shift of the bandgap to red is connected to energy losses in QDs due to carrier relaxation. Balancing the two aspects leads to an ideal position of the absorber bandgap for a single-junction solar cell at about 1.1eV.[1]

To illustrate the losses we compare the terrestrial solar spectrum (grey area) to the part, which is actually absorbed by the studied QDs (4-nm sized CdSe QDs). We assume a very high OD (3.5 at the lowest excitonic band) and the absorbed part of energy is denoted by the red region in Figure S1.

Independently of the initial photon energy, the energy of an excited electron-hole pair after relaxation within QD is about 2.12eV and the excitation energy above this value (blue area) is lost for conversion (relaxation losses). Consequently the carriers have to be extracted to electrodes, where the potential difference is typically about 0.5V. This leads to further losses denoted by cyan area (relaxation losses).

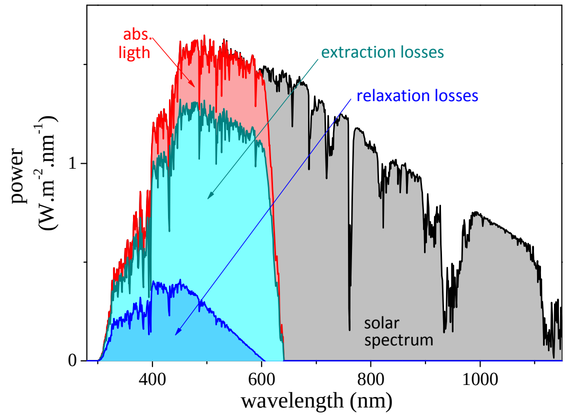

Figure S1. Solar spectrum (grey area) compared to the energy absorbed by QDs (below red curve). The absorbed energy is lost for conversion due to relaxation in QDs (blue area) and carrier extraction (cyan area).

2. Linker as a finite barrier

Effective mass approximation is a simple theory applicable presumably applicable for large QDs. The theory assumes that electron and hole in QD can be considered as "particle-in-box" problem with effective masses known from bulk material. For CdSe the electron and hole effective masses (m_{eff}) are 0.13 and 0.45, respectively.

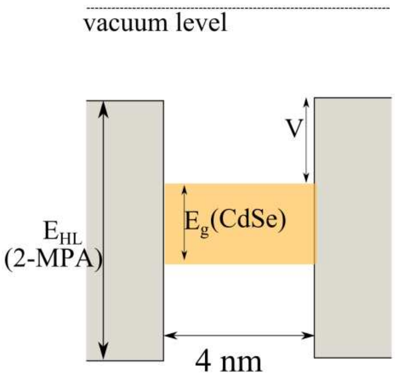

Figure S2. Scheme of potential barrier used for calculation of electron and hole lowest energy states.

Scheme S2 illustrates the calculated situation. We assume spherical CdSe QD (radius R=2 nm) with the bulk bandgap E_g (CdSe) = 1.74 eV. A potential barrier is formed by the HOMO and LUMO orbitals of the linker molecule (2-mercaptopropionic acid, HOMO-LUMO gap 8 eV).[2] We calculate the lowest electron and hole state based on the commonly used expression for the "particle-in-box" problem with finite barrier of spherical shape:

$$-cotg\left(\sqrt{\frac{2m_{eff}m_0E}{\hbar^2}}R\right) = \sqrt{\frac{V-E}{E}}\ , \qquad \text{(S1)}$$

where m_0 stands for electron mass, V for potential barrier height, and E denotes the electron/hole state energy difference from the bottom of potential well.

By using this approach we get a shift of electron state energy by about 0.5 eV and hole state energy by approx. 0.18 eV. In total we obtain the bandgap between the lowest electron and hole state to be around 2.4 eV. By taking into account the reported exciton binding energy (0.2 eV)[3] we expect the lowest exciton absorption band to be at 2.2 eV. This well reproduces the real QD optical bandgap (2.12 eV).

The calculated bandgap differs within the scale of tens of meV for different energetical alignment of linker HOMO and LUMO states. Namely, we have varied the alignment by +1eV and -1eV from symmetrical position of linker HOMO-LUMO around QD bandgap. Results are summarized in Table S1. The clear outcome of the calculations is that the energetical shift in linker orbitals +1 eV/-1eV leads to blue/red shift of the bandgap energy by about 15 meV/28 meV.

Table S1. Change in QD bandgap depending on the linker molecule HOMO and LUMO energy alignment. Symmetrical HOMO-LUMO energy alignment around QD bandgap is taken as a reference point.

HOMO-LUMO shift [eV]	confinement energy electron [meV]	hole [meV]	total bandgap [eV]	bandgap change [meV]
-1	504	180	2.424	-28
-0.5	521	179	2.440	-12
0	535	177	2.452	0
0.5	547	174	2.461	9
1	556	171	2.467	15

3. Cold electron injection analysis

The measured long-lived transient absorption (TA) kinetics (see Figure 2B, main article) were fitted by a two-exponential decay, leading to decay lifetimes and corresponding amplitudes listed in Table S2. Mean lifetimes were calculated based on the following formula:

$$\langle\tau\rangle = \frac{A_1\tau_1^2 + A_2\tau_2^2}{A_1\tau_1 + A_2\tau_2}. \qquad \text{(S2)}$$

The resulting mean lifetimes were 6.8 ns for QDs in solution, 8.5 ns for QDs on SiO_2 and 1.6 ns for the QD-ZnO sample. By calculating difference of rates, the mean lifetime of the additional decay can be determined as:

$$\frac{1}{\tau_{add}} = \frac{1}{\tau_{QD-ZnO}} - \frac{1}{\tau_{QD}}. \qquad \text{(S3)}$$

The resulting lifetime of the additional decay process in the QD-ZnO sample is 2.0 ns.

Table S2. Lifetimes and correcponsing amplitudes of two-exponential fit used to describe the long-term TA kinetics.

sample	τ_1 [ns]	A_1 [%]	τ_1 [ns]	A_1 [%]	$<\tau>$ [ns]
QD	0.6	24	7	76	6.8
QD-SiO_2	0.7	42	9	58	8.5
QD-ZnO	0.22	50	1.8	50	2.0

4. Onset rate analysis

We have analyzed the full set of data (18 measurements) by a fitting procedure, where each probe wavelength was fitted independently by equation 1 (see the main text). We observe two distinct regions of TA onset signal rate, as it can be seen from rates determined for the solution sample at various excitation wavelengths (see Figure S3).

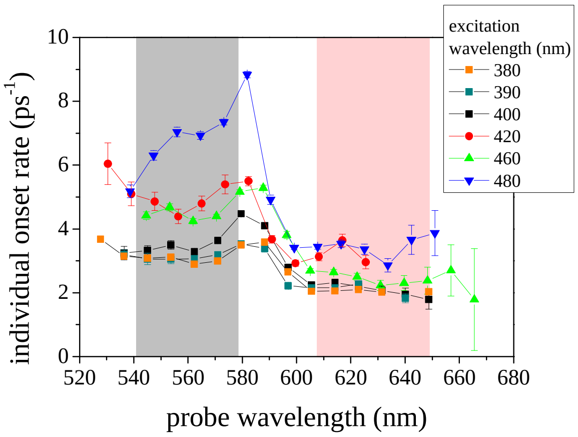

Figure S3. TA onset rates obtained for solution sample of CdSe QDs by fitting each probe wavelength. Each curve represents one excitation wavelength. Regions used for global fitting (see text for details) are marked as a shaded area.

To get as precise value of the onset rate as possible, we consequently globally fit separately the two regions (denoted as shaded area in Figure S3). We did not include the crossing region, where a transition between the two regions is present. In the global fitting (for each of the two spectral regions) both rate values (k_R and k_D) in equation 1 are shared for different probe wavelength, component

amplitudes are left free for each curve. By this fitting we obtained values in Figure 5 (main text) used for discussion of relaxation in our system.

5. Effective onset time approximation for two-step relaxation

Carrier relaxation in QD can take place either via a step-by-step relaxation through intermediate states or via a direct relaxation, which is skipping many intermediate levels – for instance by Auger type of relaxation, where electron transfers its excess energy to hole. We cannot a priori say which type of relaxation prevails in each case. A question may arise, whether using a single relaxation rate to describe the consecutive multiple-step process is a valid approximation.

To answer this concern we have numerically modeled a two-step process for the measured rates. We use real parameters for pump and probe pulse length (80 fs) and numerically model the processes depicted in inset of Figure 5. For the relaxation to the upper level (k_R) we use the experimentally measured value. Rate of relaxation to the lower level (k_F) is presumably unknown. Based on previously published data we expect the value to be about 2 ps^{-1}. We observe a good agreement with our data for the value of 3 ps^{-1}. The difference between the rates can be explained by different surface chemistry for the reported QDs (TOPO) and our samples (2-MPA).

Numerical simulation leads to an onset of TA signal, which has slightly different shape from single-exponential rise (see Figure S4). On the other hand, the difference is only minor and well below experimental noise of our data. In fact, a similar difference between curve and fit observed in Figure 4 can be traced also in fitting of our data for the lower level. This can be also taken as an indication, that the relaxation to the lower level (red region of Figure S3) is at least a two-step process. At the same time we see that it is reasonable to approximate the TA onset by a single effective rise time (see fit in Figure S4).

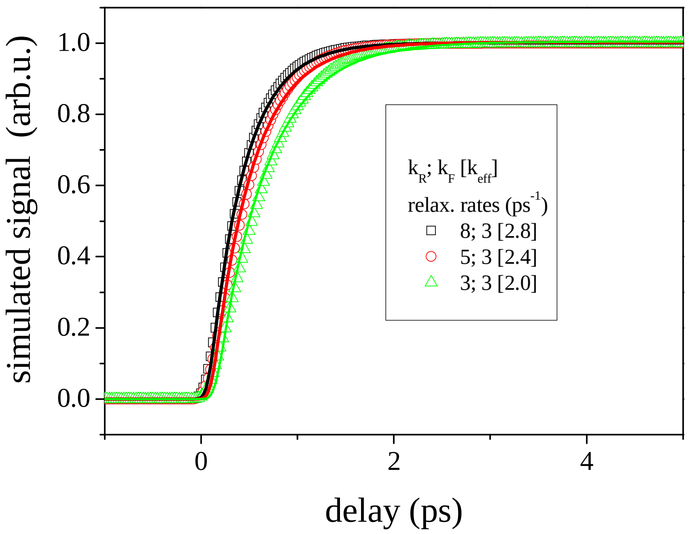

Figure S4. Simulated TA signal from a two-step modeled process (open symbols) by using the model depicted in Figure 5 (main article). The signal is compared to fit by a single effective onset rate (solid lines).

6. Pump-probe amplitude correction for losses

We use in our article analysis of the pump-probe amplitude. This implies including two main correcting factors: (i) amount of absorbed energy in the sample and (ii) losses in excitation power before the absorption.

In order to correct for the first factor, we used the spectrum of bare QDs in solution and renormalized it according to the lowest absorption band of the QD-SiO_2 and QD-ZnO samples. The renormalized curve was used to determine contribution of QDs to OD for each excitation wavelength. This enables calculation of the amount of absorbed energy in the sample ($1\text{-}10^{-OD(abs)}$).

Factor (ii) is also important, as the amplitudes of the pump-probe signal were analyzed for excitation wavelength down to 390 nm. This is already very close to the ZnO bandgap (see Figure 2) and therefore the amplitude of the QD-ZnO sample will be diminished by the ZnO absorption. We used the previously determined QD absorption spectra and subtracted it from the total spectrum to obtain losses from scattering and ZnO. The losses OD(loss) diminish the excitation by $10^{-0.5OD(loss)}$. The factor ½ takes into account the fact, that losses take place continuously throughout the ZnO layer, where QDs already absorb the light.

The limitations of the corrections can be viewed from the results of the QD-ZnO sample. At the excitation wavelength of 380 nm the QD absorption (OD of 0.03) competes with huge losses due to ZnO absorption (OD of 2.1). Here this approach is not able to provide a reasonable correction.

For both factors we assume that the resulting signal amplitude is proportional to the absorbed energy. This approach can be used, as we excite QDs with a low excitation intensity (<N> ~ 0.1)

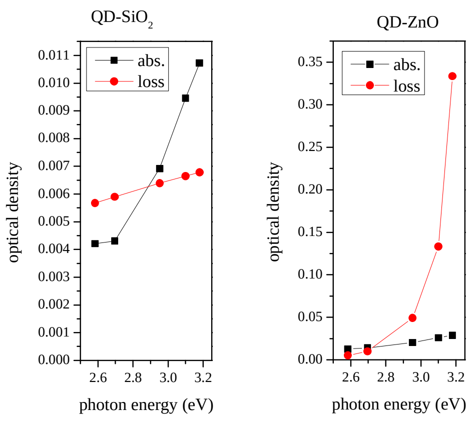

Figure S5. Optical density of samples corresponding to QD absorption (black color) and losses due to scattering and ZnO absorption (red color). The values were used to correct the amplitude of the pump-probe signal.

References

(1) Shockley, W.; Queisser, H. J. *J. Appl. Phys.* **1961**, *32*, 510.

(2) Hansen, T.; Žídek, K.; Zheng, K.; Abdellah, M.; Chábera, P.; Persson, P.; Pullerits, T. *J. Phys. Chem. Lett.* **2014**, *5*, 1157.

(3) Meulenberg, R. W.; Lee, J. R. I.; Wolcott, A.; Zhang, J. Z.; Terminello, L. J.; van Buuren, T. *ACS Nano* **2009**, *3*, 325.
